# Quantification of bacterial fluorescence using independent calibrants

**DOI:** 10.1371/journal.pone.0199432

**Published:** 2018-06-21

**Authors:** Jacob Beal, Traci Haddock-Angelli, Geoff Baldwin, Markus Gershater, Ari Dwijayanti, Marko Storch, Kim de Mora, Meagan Lizarazo, Randy Rettberg, with the iGEM Interlab Study Contributors

**Affiliations:** 1 Raytheon BBN Technologies, Cambridge, MA, United States of America; 2 iGEM Foundation, Cambridge, MA, United States of America; 3 Department of Life Sciences, Imperial College London, London, United Kingdom; 4 Synthace, London, United Kingdom; Dartmouth College, UNITED STATES

## Abstract

Fluorescent reporters are commonly used to quantify activities or properties of both natural and engineered cells. Fluorescence is still typically reported only in arbitrary or normalized units, however, rather than in units defined using an independent calibrant, which is problematic for scientific reproducibility and even more so when it comes to effective engineering. In this paper, we report an interlaboratory study showing that simple, low-cost unit calibration protocols can remedy this situation, producing comparable units and dramatic improvements in precision over both arbitrary and normalized units. Participants at 92 institutions around the world measured fluorescence from *E. coli* transformed with three engineered test plasmids, plus positive and negative controls, using simple, low-cost unit calibration protocols designed for use with a plate reader and/or flow cytometer. In addition to providing comparable units, use of an independent calibrant allows quantitative use of positive and negative controls to identify likely instances of protocol failure. The use of independent calibrants thus allows order of magnitude improvements in precision, narrowing the 95% confidence interval of measurements in our study up to 600-fold compared to normalized units.

## Introduction

Fluorescent reporters are one of the most commonly used methods for quantifying the behavior of natural or engineered cells. Despite the popularity of fluorescent reporters and the well-established availability of fluorescence calibration standards (e.g., [1, 2]), however, measurements of fluorescence are still typically reported in relative units, generally either completely arbitrary (“a.u.”) or else normalized against some control cell sample (e.g., the prior standardization recommendations in RPU [3], ratiometric [4], REU [5]). For example, in the January 2016 issues of *Nature* and *Science* alone, there were ten articles published that used plate readers or flow cytometry to quantify cellular fluorescence. Of these articles, five used normalized units, five used arbitrary units, and none determined units using an independent calibrant. Such use of relative units runs counter to typical scientific practice, as comparable units of measurement are generally considered foundational to the scientific method.

The lack of comparable units is even more problematic in synthetic biology, an engineering field based on the predictable manipulation of genetic components [6, 7]. Fluorescence is simple to measure across a wide dynamic range of values, and thus synthetic biologists commonly utilize fluorescence to characterize and debug the devices and systems that they engineer. Yet just like other biological researchers, they still largely use arbitrary or relative units for fluorescence measurements. For example, in the January and Febuary 2016 issues of *ACS Synthetic Biology*, there were ten articles published that used plate readers or flow cytometry to quantify cellular fluorescence, of which five used normalized units, four used arbitrary units, and only one used an independent calibrant. Every mature field of engineering, however, from carpentry to computer science, from chemical processing to aerospace engineering, depends critically on shared units of measurement to enable the reliable composition of methods, models, and components developed by disparate individuals and organizations. Moreover, approaches to fluorescence calibration have already been solidly established in other fields (e.g., [1, 2]), though standard reference materials are still an evolving question. We therefore argue that for synthetic biologists to be able to effectively develop genetic systems, it is important to develop and promote good measurement practices with widely-tested protocols for obtaining comparable units of fluorescence.

Are relative fluorescent units, however, actually problematic when dealing with measurements of biological cells? Perhaps researchers tend to use similar arbitrary units, or perhaps cellular controls vary little enough that normalized units are sufficiently precise? Complementarily, perhaps obtaining and using an independent calibrant is too difficult, unreliable, or costly relative to the value of reproducible units? To answer these questions (and also to promote the use of good measurement practices), we organized a large-scale interlaboratory study involving synthetic biologists participating in the 2016 International Genetically Engineered Machine (iGEM, http://igem.org) competition at 92 institutions around the world. Each team of contributors measured three engineered test plasmids, plus positive and negative controls, in *E. coli*. Two simple, low-cost unit calibration protocols were tested, a protocol for bulk measurements (e.g., plate readers) based on fluorescein and colloidal silica and a protocol for flow cytometers based on fluorescent beads.

The results of this study are clear and unambiguous: first, relative fluorescent units create a massive and unnecessary uncertainty in fluorescence measurements, and second, independent calibrants can readily address this problem for both bulk fluorescence and flow cytometry. Critically, we find that measurement in standard units enables principled quantitative filtering of unreliable data using the values of process control samples. The combined result of calibration and calibration-enabled filtering is a dramatic improvement in precision: the 95% confidence interval of measurements in our study is narrowed by more than four orders of magnitude over arbitrary units and up to 600-fold compared to normalized units.

## Results

**Fig 1:**
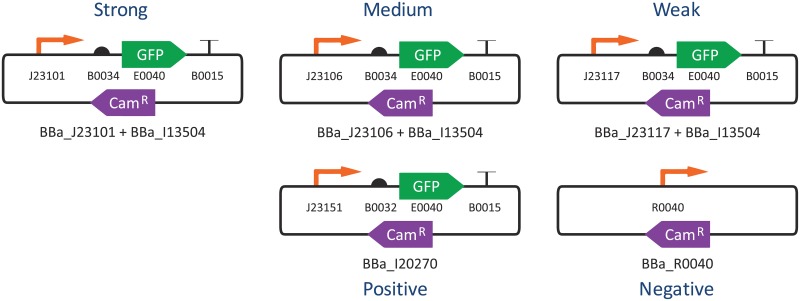
Constitutive fluorescence constructs measured in the 2016 iGEM Interlab Study, diagrammed using standard SBOL Visual symbols [9].

To study fluorescence measurement, we organized an interlaboratory study in association with the 2016 International Genetically Engineered Machine (iGEM) competition, for which 92 teams signed up to participate. All participants were provided with a collection of five engineered genetic constructs (Fig 1)—three test constructs to express green fluorescent protein (GFP) at “strong”, “medium”, and “weak” levels, plus positive and negative controls (full details in Supporting Information S1 File). The three test constructs couple a close variant of the GFP expression cassette from the measurement kit developed in [3] with promoters from one of the most widely used bacterial constitutive promoter libraries, selected to provide a wide range of expression at previously reported relative strengths of 0.70, 0.47, and 0.06 respectively [8]. Both the “strong” and positive control promoters appear in [3] as well, while the negative control is a commonly used promoter with no coding sequence to regulate.

Participants transformed *E. coli* (DH5-alpha or TOP10 strains if available) with these constructs, cultured overnight, then diluted to a target OD and incubated for six hours. Study participants were provided with two protocols for measuring fluorescence with reference to an independent calibrant, one for bulk fluorescence measurements (e.g., plate reader, spectrofluorimeter) and one for flow cytometry. Each team was asked to measure fluorescence of all constructs with one or both of the calibration protocols, using two biological replicates for bulk fluorescence or three for flow cytometry.

For bulk fluorescence measurements, participants were asked to report in units of *μ*M FITC/OD, i.e., molarity of fluorescein isothiocyanate (FITC) normalized by optical density (OD), using two provided independent calibrants. FITC is a fluorescent material that is relatively cheap, stable, and easy to work with, as well as having fluorescent excitation and emission spectra that closely match GFP. To convert from arbitrary fluorescence units to *μ*M FITC, participants were provided with a sample containing a defined concentration of FITC, from which to create a standard fluorescence curve via serial dilution, based on previously established fluorescence calibration methods [1].

Absorbance at 600 nm is frequently used as a measure of cell density and is widely used to normalize fluorescence measurements relative to the number of cells in a population. However, the observed change in absorbance is due to scattering of the incident light by cells, rather than chromophoric absorption. Its relationship to cell density thus depends on path length (and therefore instrument configuration) and is only linear at low values where multiple scattering is not significant [10]. Colloidal silica has similar scattering properties, and is also cheap, stable, and easy to work with. To convert from absorbance to a comparable OD value, participants were thus provided with a sample of colloidal silica, and asked to measure samples of silica and water. Absorbance measurements from cell samples of the same volume and container shape can then be normalized by comparison against measurements from a reference spectrophotometer. See Materials and methods for complete details.

For per-cell fluorescence measurements with flow cytometry, participants were asked to report in units of Molecules of Equivalent FLuorescein (MEFL). For this purpose, participants were asked to obtain and measure a sample of SpheroTech RCP-30-5A Rainbow Calibration Particles, a previously established calibration material for flow cytometry [2, 11]. This material comprises a mixture of fluorescence beads with several distinct known levels of fluorescence. When measured in a flow cytometer, these produce a histogram with sharp peaks whose locations determine the unit conversion factor. See Materials and methods for complete details.

### Data collection and processing

Of the 92 participating teams, 72 were ultimately able to contribute data to the experiment, producing a total of 65 bulk fluorescence data sets (mostly gathered with plate readers, but including some spectrophotometer data as well) and 14 flow cytometry data sets (seven teams contributed both bulk fluorescence and flow cytometry data). Data sets were reported by filling in a provided spreadsheet and protocol form, included as Supporting Information S2, S3, S4 and S5 Files.

The final corrected mean and standard deviation values measured for each data set are summarized in Supporting Information S1 and S2 Tables, along with the unit conversion factors computed from the calibration process for each data set (unprocessed source data is provided in S3 and S4 Tables). In addition, responses to the study’s protocol forms are included as Supporting Information S5 and S6 Tables.

In analysis, we considered three possible treatments of the data in Supporting Information S1 and S2 Tables:

- conversion to comparable units by comparison with independent calibrant measurements (details of the conversion factor calculations are provided in Methods and materials),
- normalization to a reference construct, implemented by dividing the fluorescence of each sample by the fluorescence of the positive control from the same replicate, and
- filtering to remove data sets where the protocol might have failed by “sanity checking” the values of positive and negative controls.

Filtering takes advantage of the comparability of calibrated unit measurements and the independence of process controls. Since the scale of measurements is fixed, it is possible to establish expectations of “reasonable” values for positive and negative controls. Many of the ways that problems can occur (e.g., bad reagents, mistakes in protocol execution, instrument problems) are likely to affect both these process controls and the experimental samples, so unusual values in a data set’s controls indicate a significant likelihood that problems have occurred such that the entire data set should be discarded. Conversely, controls with values in an expected range can increase confidence by at least ruling out those classes of experimental problems that would also affect the controls.

Importantly, removing data sets with aberrant process controls is a quite different sort of procedure than statistics-based removal of outliers from experimental data. The removal of experimental outliers is inherently more fragile, as it can only be based on a numerical model of the experimental data itself and cannot separate factors that may be affecting that data. Removal of data sets with aberrant process controls, on the other hand, is done entirely without reference to the values of the experimental data, and should based only on observed quantities that are not the subject of experimental study (e.g., in this study, fluorescence from the control constructs). As such, the only data sets to be removed are those that can be positively identified as affected by an interfering factor that is explicitly not under study. Thus, unlike outlier removal, process control filtering should not have any significant affect on the statistical distribution of data for which processes have been executed correctly.

In applying process control filtering in our analysis, we chose a relatively permissive filter, excluding only those data sets for which:

- the positive control has extremely high or low fluorescence (more than 3-fold difference from the median value of 3.02 *μ*M FITC/OD bulk, 3.8e4 MEFL flow),
- the negative control is strongly fluorescent (greater than 0.5 *μ*M FITC/OD bulk, 5000 MEFL flow), or
- the negative control has a significantly negative value, which should be impossible (less than −0.01 *μ*M FITC/OD, 0 MEFL flow)

Note that this rubric also necessarily excludes data sets in which all negative or all positive controls are missing. In total, filtering using these criteria retains 30 of the 65 bulk fluorescence data sets (exclusions: 3 missing controls, 14 extreme-valued positive controls, 2 high negatives, 3 low negatives, and 13 with problems in both positive and negative controls) and 6 of the 14 flow cytometry data sets (exclusions: 3 missing controls, 4 extreme-valued positive controls, and 1 high negative). Excluding slightly more than half of each data set indicates a relatively high rate of failure, which emphasizes the number of ways in which synthetic biology experiments can go wrong and the importance of comparable units for identifying and filtering out potential issues of this sort, as will be demonstrated below. We also note that the results we present are not particularly sensitive to the filtering thresholds chosen (See Supporting Information S7 Table).

Since filtering based on positive and negative process controls requires calibration and normalization produces the same values irrespective of unit, these three possible treatments can be combined to yield a total of five ways of interpreting the data from this study: arbitrary unit data, calibrated unit data, normalized data, filtered (calibrated) data, and normalized and filtered (calibrated) data. We will evaluate the benefits of independent calibrants by comparing these five data collections, computing geometric statistics as distributions of gene expression are typically log-normal [12].

### Fluorescence levels and precision

**Fig 2:**
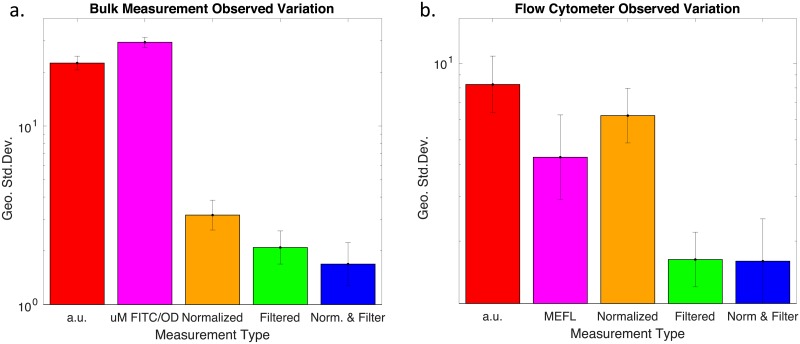
Precision effects of calibration for (a) bulk fluorescence and (b) flow cytometry, showing geometric standard deviation over the values reported from different laboratories (geometric mean over test devices ±1 geometric standard deviation).

**Fig 3:**
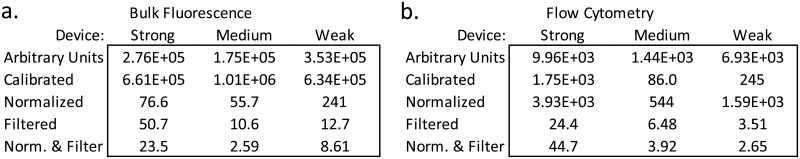
Fold range of 95% confidence interval vs. data treatment for (a) bulk fluorescence and (b) flow cytometry.

The effect of the different data treatments on precision of fluorescence measurements is reported in Fig 2. For each treatment, we compute the geometric standard deviation of measurements for the “strong”, “medium”, and “weak” devices, then compute the geometric mean and standard deviation of these values. Additional details for each device and the implications of geometric standard deviation are provided in Fig 3, which shows the size of the 95% confidence interval for each device under each data treatment, i.e., the fold range of two geometric standard deviations up and down from the measured values. The confidence interval is a useful measure of the pragmatic implications of precision, as it indicates the degree of certainty that can be readily established for the value of a biological parameter, based on measurements of fluorescence.

For bulk measurements of fluorescence, the geometric standard deviation of arbitrary unit measurements is extremely high: approximately 20-fold standard deviation implies a confidence interval over five orders of magnitude in size, meaning that measurements provide essentially no information about the true value of fluorescent expression. Calibration to units of *μ*M FITC/OD does not improve the situation, but its range is due to a small collection of strong outliers rather than a relatively even spread of values. When data sets with problematic controls are filtered out, these outliers disappear as well, yielding fairly tight distributions with a mean 2.1-fold standard deviation. Normalized measurements are also much better than arbitrary unit measurements, but still can be significantly improved (*p* = 0.03) by the use of calibrated units to enable process control filtering, improving the mean standard deviation from 3.2-fold to 1.7-fold. In sum, we find that calibration and process control filtering improve quantification of biological parameters via bulk measurement by approximately four orders of magnitude improvement in confidence interval compared to arbitrary unit measurements and one order of magnitude improvement over normalized measurements.

Flow cytometry shows similar patterns of improvement from calibration. The baseline geometric standard deviation for arbitrary units is not as high as for bulk measurement, likely due to a greater degree of uniformity in flow cytometer instrument design. With this data, calibration to units of MEFL provides more improvement than with bulk fluorescence (though not significantly so, *p* = 0.07), while normalization does not help as much (and is also not significant, *p* = 0.24). As with bulk fluorescence, however, dramatic improvements are seen when calibrated units are used to filter out data sets with problematic controls, providing significant improvement (*p* = 0.02, *p* = 0.008) to a mean standard deviation of 1.7-fold for both absolute and normalized units. In sum, we find that calibration and process control filtering improve quantification of biological parameters via flow cytometry by approximately three orders of magnitude improvement over arbitrary unit measurements and two orders of magnitude improvement over normalized measurements.

**Fig 4:**
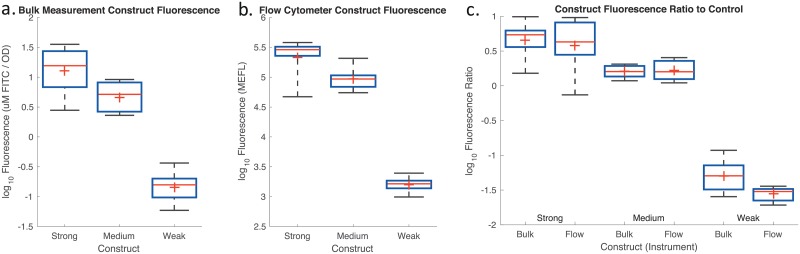
Measured fluorescence of test devices for (a) bulk fluorescence, (b) flow cytometry, and (c) both types of instruments, normalized against the positive control. In each box, red plus indicates mean, red line indicates median, top and bottom edges indicate 25th and 75th percentiles, and whiskers extend from 9%–91%.

Finally, we further confirm the validity of the calibration and process control filtering by comparing the distribution of calibrated values computed for the three devices by the two different modalities of measurement, as reported in Fig 4 (accuracy in measurement of a previously indeterminate value is validated by comparison of different modalities of measurement). Since the units are different for bulk fluorescent measurements and per-cell measurements in flow cytometry, their absolute values cannot be directly compared, but we note that the values of the strong, medium, and weak constructs show closely similar patterns in relative levels. Comparison of values normalized against the positive control confirms this similarity: the strong and medium devices have extremely similar geometric means, having only 1.2-fold and 1.05-fold differences respectively, while the weak devices show a slightly larger 1.8-fold difference in geometric means (possibly due to the greater dynamic range typical in flow cytometers). Finally, we note that the observed fluorescence levels are also at least roughly consistent with the prior single-group characterization reported for these devices [8], though direct comparison cannot be made due to differences in protocol and expression context.

## Discussion

The results of this study illustrate the critical importance of using independent calibrants in the measurement of cellular fluorescence. It is unsurprising that measurement precision can be greatly increased by using the same units rather than arbitrary units—though it is notable that the difference between instruments is not small, but can span many orders of magnitude. Even after eliminating the top and bottom 10% of unit conversion factors, bulk fluorescence measurements in this study still span a 1850-fold range in the unit conversion factors for fluorescence and a 135-fold range for OD, while flow cytometry fluorescent unit conversion factors span a 10.7-fold range (and the tighter range on flow cytometry is likely due only to the protocol instructions on how to adjust measurement range in support of calibration).

More significantly, our results show that normalization against a “known construct” control is highly unreliable as a means of reducing uncertainty in measurement. Biological experiments are well known to be challenging to execute consistently and to replicate, and many of the challenges that can be encountered are likely to affect cellular controls as well, thus making it difficult to determine whether problems in protocol execution have occurred or not. This is borne out in our study by the fact that normalization against the positive control still resulted in measurements with high degrees of uncertainty. In our data, the effect was less pronounced for bulk fluorescence than it was for flow cytometry, but there is no reason to believe this difference is actually systematic, as opposed to an artifact of the particular collection of outlier failures that dominate the uncertainty in each of the two data sets. Moreover, the protocols and constructs used in our study are quite simple and well-established: the degree of uncertainty is likely to become much higher when dealing with more complex or subtle systems and experiments.

Failures can still happen with independent calibrants, of course, but there is much less chance that a failure of the calibrant will be correlated with a failure in the experimental protocol. This is particularly the case with simple and stable calibrant materials such as those used in this study. Furthermore, as we have seen in this study, using two independent sets of controls—e.g., independent calibrants and cellular controls—can greatly reduce measurement uncertainty by allowing the cellular controls to serve as process controls rather than calibrants, thus enabling principled identification and exclusion of dubious data sets. Given the scales of uncertainty involved, we thus argue that the use of independent calibrants is of critical importance for fluorescence-based quantification of the properties of biological cells. This is even more critical in an engineering context, as engineering cannot be effective without the ability to predictably and routinely combine methods, models, and components developed by different people and organizations.

There is, however, still need for further improvement in the precision that can be achieved in measurement between laboratories. We note in particular that the “strong” device exhibited a much greater degree of outliers than either of the other two devices, and inspection of data sets revealed that for many teams its absorbance values were much lower than the controls or other devices, thus suggesting there may be culturing issues associated with that particular construct. There are known spectral issues in fluorescence measurement that can potentially be addressed by standardization of filters or correction formulae. The protocol for OD calibration is also not yet satisfactory as an approach to normalizing for cell population size, given its dependence and some of the known issues in use of absorbance as a proxy for cell count (e.g., [10]). Finally, it is not yet clear what degree of precision is an appropriate target to aim for in dealing with quantification of populations of biological cells. Already, however, the degree of precision achieved in this study indicates that cheap and readily accessible independent calibrants can enable measurement of sufficient precision to support a much greater degree of replication, sharing, and composition than is currently practiced in the engineering of biological organisms.

## Materials and methods

### Calibration materials

Every participating team was provided with a measurement kit comprising seven tubes (five with plasmid DNA and two with calibrants):

- Plasmid DNA (100 pg/uL in 10 uL of Buffer EB)
  - Test Device 1: J23101.B0034.E0040.B0015 in pSB1C3
  - Test Device 2: J23106.B0034.E0040.B0015 in pSB1C3
  - Test Device 3: J23117.B0034.E0040.B0015 in pSB1C3
  - Positive Control Device: I20270 in pSB1C3
  - Negative Control Device: R0040 in pSB1C3
- FITC Standard: one tube with 5.00e-8 M dried down FITC
- LUDOX-HS30 (Sigma-Aldrich): one tube with 30% colloidal silica suspended in 1mL of water

FITC standard tubes were prepared by first combining 165.6 mg of FITC (Sigma F4274) powder with 0.1 L Dimethylformamide (DMF) to produce a 4.253 mM solution. Each tube received 11.76 uL of this solution (5.00e-8 M FITC), which was then vacuum dried for shipping. Resuspension in 1 mL PBS would thus produce a solution with initial concentration of 50 *μ*M FITC.

### Culturing and measurement protocols

The protocol for plate readers, exactly as supplied to each participating team, is listed in Supporting Information S2 and S4 Files. Likewise, the protocol for flow cytometers, exactly as supplied to each participating team, is listed in Supporting Information S3 and S5 Files.

### Computation of unit conversion factors

#### Absorbance to optical density

For each data set, corrected absorbance was computed by subtracting the average absorbance measured for water from the average absorbance measured for LUDOX-HS30. This was compared to a reference value obtained via a spectrophotometer (for which path length and vessel geometry are not variable). The conversion factor from absorbance to standardized optical density was then taken to be the corrected measurement from the reference spectrophotometer divided by the corrected measurement for each data set.

#### Fluorescence serial dilution model

For each data set, corrected fluorescence was first computed by subtracting the mean fluorescence of 0 FITC samples from the mean fluorescence of the rest of the samples. Next, as the serial dilution spans three orders of magnitude, it was frequently the case that a portion would be saturated high or low. Saturated portions of the serial dilution (and other dubious points) were thus removed by eliminating any point whose difference from the next point was not close enough to the expected slope: fluorescence should decrease by 2 at each step, so values with a decrease less than 1.5 or greater than 3 were removed. Finally, the slope was fit against a serial dilution model including systematic pipetting error, such that the fluorescence for each dilution stage is:

$$\begin{array}{c} f_{n}=M\cdot \left(0.5+\beta \right)\cdot {\left(0.5-\beta \right)}^{n-1} \end{array}$$ (1)

where *f*_*n*_ is the fluorescence for the *n*th dilution, *β* is the systematic pipetting error, and *M* the initial molarity of the FITC standard (in this case 5 *μ*M). The *β* parameter of the serial dilution model and the *α* conversion factor from arbitrary units to *μ*M FITC were then simultaneously fit against non-excluded data points to minimize sum squared error:

$$\begin{array}{c} \epsilon =\sum |\log\left(\alpha \frac{f_{n}}{x_{n}}\right){|}^{2} \end{array}$$ (2)

where *ϵ* is sum squared error of the fit and *x*_*n*_ is the mean corrected arbitrary unit value of the *n*th titration stage. The *α* value thus computed provides the unit conversion factor from a.u. to *μ*M FITC.

#### Fluorescent beads

For flow cytometry measurements, SpheroTech RCP-30-5A beads were used as the reference material. A sample of this material is a mixture of particles with eight levels of fluorescence, which should appear as up to eight peaks (typically some are lost to saturation on the instrument). Teams reported the arbitrary unit value of each visible peak, and the conversion factor was then computed as the average ratio of arbitrary unit to calibration value provided by the manufacturer for FITC for Lot AA01, AA02, AA03, AA04, AB01, AB02, AC01, and GAA01-R. Note that lot was not recorded, and thus some differences in value for flow cytometry data (up to around 10%) may be due to differences in the calibration values of between lots.

## Supporting information

S1 File DNA constructs. SBOL 2 formatted file [13] containing DNA constructs for the 2016 iGEM Interlab Study.
(XML)

S2 File Bulk fluorescence protocol form. Detailed protocol specification and reporting form provided for plate readers for the 2016 iGEM Interlab Study.
(PDF)

S3 File Flow cytometer protocol form. Detailed protocol specification and reporting form provided for flow cytometers for the 2016 iGEM Interlab Study.
(PDF)

S4 File Bulk fluorescence measurement form. Detailed measurement reporting spreadsheet provided for plate readers for the 2016 iGEM Interlab Study.
(XLS)

S5 File Flow cytometer measurement form. Detailed measurement reporting spreadsheet provided for flow cytometers for the 2016 iGEM Interlab Study.
(XLSX)

S1 Table Bulk fluorescence summary data. Datasets analyzed for plate readers for the 2016 iGEM Interlab Study.
(CSV)

S2 Table Flow cytometer summary data. Datasets analyzed for flow cytometers for the 2016 iGEM Interlab Study.
(CSV)

S3 Table Unprocessed bulk fluorescence data. Unprocessed data for plate readers for the 2016 iGEM Interlab Study, summarized in S1 Table.
(XLSX)

S4 Table Unprocessed flow cytometer data. Unprocessed data for flow cytometers for the 2016 iGEM Interlab Study, summarized in S2 Table.
(XLSX)

S5 Table Bulk fluorescence protocol form responses. **Response information from the protocol specification and reporting form for bulk fluorescence for the 2016 iGEM Interlab Study**. Only technical entries for the form are included, and team names have been replaced by numbers corresponding to the numbers in the summary data table. Two protocol form entries are missing, one from a team that returned their form manually due to Internet censorship in China, and one from a team that did not return any form.
(XLSX)

S6 Table Flow cytometer protocol form responses. **Response information from the protocol specification and reporting form for flow cytometers for the 2016 iGEM Interlab Study**. Only technical entries for the form are included, and team names have been replaced by numbers corresponding to the numbers in the summary data table.
(XLSX)

S7 Table Effect of filter threshold choice. Datasets retained and geometric standard deviation under perturbation of process control filtering thresholds.
(CSV)

## References

[1] Vogt RF Jr, Marti GE, Zenger V. Quantitative fluorescence calibration: a tool for assessing the quality of data obtained by fluorescence measurements In: Standardization and Quality Assurance in Fluorescence Measurements I. Springer; 2008 p. 3–31.

[2] Hoffman RA, Wang L, Bigos M, Nolan JP. NIST/ISAC standardization study: Variability in assignment of intensity values to fluorescence standard beads and in cross calibration of standard beads to hard dyed beads. Cytometry Part A. 2012;81(9):785–796. doi: 10.1002/cyto.a.22086 10.1002/cyto.a.22086 22915363

[3] Kelly JR, Rubin AJ, Davis JH, Ajo-Franklin CM, Cumbers J, Czar MJ, et al Measuring the activity of BioBrick promoters using an in vivo reference standard. Journal of Biological Engineering. 2009;3(4). doi: 10.1186/1754-1611-3-4 19298678 10.1186/1754-1611-3-4 PMC2683166

[4] Yordanov B, Dalchau N, Grant PK, Pedersen M, Emmott S, Haseloff J, et al A computational method for automated characterization of genetic components. ACS synthetic biology. 2014;3(8):578–588. doi: 10.1021/sb400152n 24628037 10.1021/sb400152n

[5] Stanton BC, Nielsen AA, Tamsir A, Clancy K, Peterson T, Voigt C. Genomic mining of prokaryotic repressors for orthogonal logic gates. Nature Chemical Biology. 2014;10(2):99–105. doi: 10.1038/nchembio.1411 24316737 10.1038/nchembio.1411 PMC4165527

[6] Purnick PE, Weiss R. The second wave of synthetic biology: from modules to systems. Nature reviews Molecular cell biology. 2009;10(6):410–422. doi: 10.1038/nrm2698 19461664 10.1038/nrm2698

[7] Cheng AA, Lu TK. Synthetic biology: an emerging engineering discipline. Annual review of biomedical engineering. 2012;14:155–178. doi: 10.1146/annurev-bioeng-071811-150118 22577777 10.1146/annurev-bioeng-071811-150118

[8] Anderson JC. Anderson promoter collection; Retrieved Oct. 30, 2015. http://parts.igem.org/Promoters/Catalog/Anderson.

[9] Quinn JY, Cox RS 3rd, Adler A, Beal J, Bhatia S, Cai Y, et al SBOL Visual: A Graphical Language for Genetic Designs. PLoS Biol. 2015;13(12):e1002310 doi: 10.1371/journal.pbio.1002310 26633141 10.1371/journal.pbio.1002310 PMC4669170

[10] Stevenson K, McVey AF, Clark IB, Swain PS, Pilizota T. General calibration of microbial growth in microplate readers. Scientific reports. 2016;6:38828 doi: 10.1038/srep38828 27958314 10.1038/srep38828 PMC5153849

[11] SpheroTech. Measuring Molecules of Equivalent Fluorescein (MEFL), PE (MEPE) and RPE-CY5 (MEPCY) using Sphero Rainbow Calibration Particles. SpheroTech; 2001. SpheroTechnical Notes: STN-9, Rev C 071398.

[12] Beal J. Biochemical complexity drives log-normal variation in genetic expression. Engineering Biology. 2017;1(1):55–60. doi: 10.1049/enb.2017.0004

[13] Roehner N, Beal J, Clancy K, Bartley B, Misirli G, Grünberg R, et al Sharing structure and function in biological design with SBOL 2.0. ACS synthetic biology. 2016;5(6):498–506. doi: 10.1021/acssynbio.5b00215 27111421 10.1021/acssynbio.5b00215

