# Supplementary material for: Quantification of bacterial fluorescence using independent calibrants
Source: PLoS One. 2018 Jun 21;13(6):e0199432. doi: 10.1371/journal.pone.0199432 (PMC6013168; doi:10.1371/journal.pone.0199432)
Supplement: S2 File — Detailed protocol specification and reporting form provided for plate readers for the 2016 iGEM Interlab Study. (PDF) [file pone.0199432.s002.pdf]

2016 InterLab Protocol

Every team that participates in the 2016 iGEM InterLab study needs to fill in this form. Fill this in as best as you can. It's primarily based on E. coli work, but most questions can apply to other organisms as well.

If you have any questions or problems filling in this form, please email us at measurement at igem dot org.

* Required

1. **Team name** *

   Please enter your team name below. Please make sure it's the same as your official team name, including any hyphens or other characters.

   ....

2. **InterLab Study wiki page** *

   Please provide the direct link to your team's wiki page for the InterLab study. As explained in the InterLab requirements, all teams must have a specific page for the InterLab study on their wiki.

   ....

3. **Individuals responsible for conducting InterLab study** *

   Please list everyone involved with creating the devices, measuring them, and processing the data. Please indicate which role each person filled.

   ....
   ....
   ....
   ....
   ....

4. **Corresponding email** *

   ....

5. **List anyone else who should be credited, e.g., in a publication based on this data**

   ....
   ....
   ....
   ....
   ....

Chassis and Safety Information

6. **What chassis did you use?** *
Please note the cell type you used to conduct the InterLab Study. Be as specific as possible, organism_strain. Example: E. coli K-12 DH5-alpha (not just E. coli)
Mark only one oval.

- ( ) Escherichia coli Top10
- ( ) Escherichia coli DH5alpha
- ( ) Other: ....

7. **What Biosafety Level is your chassis?** *
Remember - BSL3 and 4 organisms are banned in iGEM. If you have questions about safety, please email safety at igem dot org!
Mark only one oval.

- ( ) BSL1
- ( ) BSL2
- ( ) I'm not sure

8. **What PPE did you utilize during your experiments (from cloning through to measuring the devices)?** *
Note any Personal Protective Equipment (PPE) used during this process. This could include gloves, lab coats, long pants, etc.

....
....
....
....
....

Calibration Protocol

9. **What instrument did you use during your measurements?** *
Check all that apply.

- [ ] plate reader
- [ ] spectrophotometer
- [ ] fluorimeter
- [ ] Other: ....

10. **Please provide the brand and model of your instrument** *
(e.g., Tecan Infinite 200)

....

OD600 Reference Point

You will use LUDOX-S30 as a single point reference to obtain a ratiometric conversion factor to transform your absorbance data into a standard OD600 measurement. YOU MUST THEREFORE TURN OFF PATHLENGTH CORRECTION. To measure your standard LUDOX Abs600 you must use the same cuvettes, plates and volumes (suggestion: use 100 uL for plate reader measurement and 1 mL for

spectrophotometer measurement) that you will use in your cell based assays.

If using plates prepare a column of 4 wells with 100 uL 100% LUDOX and 4 wells containing 100 uL H2O. Repeat the measurement in all relevant modes used in your experiments (e.g. settings for orbital averaging).

If using a cuvette, you will only have enough material for a single measurement, but repeat the reading multiple times. Use the same cuvette to measure the reference with H2O (this value will be subtracted by the instrument to give the OD600 reading).

Instrument setting

11. **Did you use pathlength correction during measurement?** *

Mark only one oval.

- ( ) Yes
- ( ) No

12. **Number of flashes per well** *

....

13. **Orbital averaging (mm)** *

....

14. **What temperature setting did you use during the measurement?**

....

Measurement

15. **Steps** *

Please check off each step that you followed. If you did anything differently or extra, please note that in the "Other" box.

Check all that apply.

- [ ] Prepare your 96 well plate or cuvettes
- [ ] Add 100 µl LUDOX 100 % into wells A1, B1, C1, D1 (or 1 mL LUDOX 100% into a cuvette)
- [ ] Add 100 µl of H2O into A2, B2, C2, D2 (or 1 mL H2O into a cuvette)
- [ ] Measure absorbance 600 nm of all samples in all standard measurement modes in instrument
- [ ] Import data into "Abs600" blue cells in provided Excel calibration sheet
- [ ] Other: ....

Protocol FITC Fluoresence standard curve

In this protocol, you will prepare a dilution series of FITC in 4 replicates and measure the fluorescence in a 96 well plate in your plate reader or individually in cuvettes in a fluorimeter. This will allow your measurements to be converted from arbitrary fluorescence into units of "Molecules of Equivalent Fluorescein" (MEFL).

Before beginning this protocol ensure that you are familiar with the GFP settings and measurement modes of your instrument. The settings that you use should be exactly the same ones that you will use when measuring your cells (if you change them you will not be able to use this standard curve). If you aren't absolutely certain which you will use, it can be a good idea to repeat the measurement a number of times with different settings. You will then have a series of standard curves to choose from without needing to redo this protocol. Most important, it is necessary to use a number of settings that affect the sensitivity (principally gain and/or slit width). Be sure to also consider other options (orbital averaging, top/bottom optics). As before, TURN OFF path length correction if available.

Instrument setting

16. **Did you use pathlength correction during measurement?** *
Mark only one oval.
- ( ) Yes
- ( ) No

17. **Number of flashes per well** *

18. **What gain setting did you use?** *

19. **Did you use a filter on your instrument?** *
Mark only one oval.
- ( ) Yes
- ( ) No

20. **If you used a filter, what light wavelengths did it pass?**
(e.g., 530 nm / 30 nm bandpass)

21. **Emission wavelength** *

22. **Excitation wavelength** *

23. **Fluorescence reading** *
Mark only one oval.
- ( ) top optic
- ( ) bottom optic
- ( ) Other: ________

24. **What temperature setting did you use during the measurement?**

Measurement

25. **Part 1: Prepare the FITC stock solution** *

Note: it is important that the FITC is properly dissolved. To check this after the incubation period pipetted up and down – if any particulates are visible in the pipette tip continue to incubate overnight. Please check off each step that you followed. If you did anything differently or extra, please note that in the "Other" box.

Check all that apply.

- [ ] Spin down FITC stock tube to make sure pellet is at the bottom of tube.
- [ ] Prepare 2x FITC stock solution (500 μM) by resuspending FITC in 1ml of 1x Phosphate Buffer Saline (PBS).
- [ ] Incubate the solution at 42°C for 4 hours.it is important that the FITC is properly dissolved. To check this after the incubation period pipetted up and down – if any particulates are visible in the pipette tip continue to incubate overnight.
- [ ] Dilute the 2x FITC stock solution in half to make a 1x FITC solution (final concentration is 250 μM).
- [ ] Other:

Illustration of serial diluation samples in 96 well plate or cuvettes: value decreases by 2-fold with each column (50% in column 2, 25% in column 3, 12.5% in column 4, etc.)

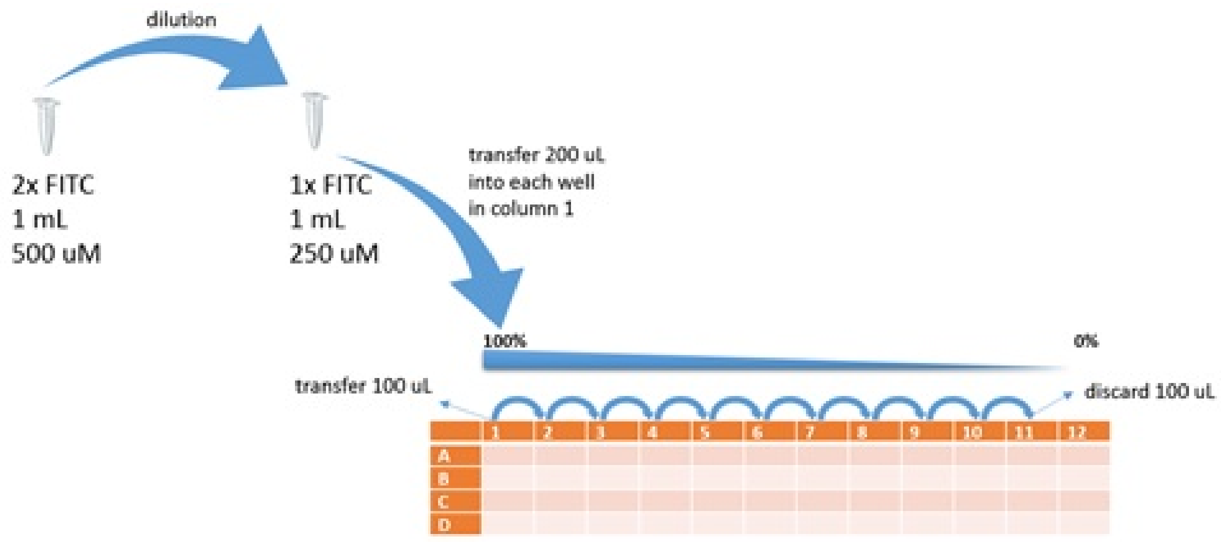

26. **Part 2 (Plate Reader): Prepare the serial dilutions of FITC**

If you are using a plate reader, please use this protocol. Note: Accurate pipetting is essential. Serial dilutions will be performed across columns 1-11. COLUMN 12 MUST CONTAIN PBS BUFFER ONLY. Initially you will setup the plate with the FITC stock in column 1 and an equal volume of 1xPBS in columns 2 to 12. You will perform a serial dilution by consecutively transferring 100 uL from column to column with good mixing. Please check off each step that you followed. If you did anything differently or extra, please note that in the "Other" box.

Check all that apply.

- [ ] Add 100 µl of PBS into wells A2, B2, C2, D2....A12, B12, C12, D12
- [ ] Add 200 µl of FITC stock solution into A1, B1, C1, D1
- [ ] Transfer 100 µl of FITC stock solution from A1 into A2.
- [ ] Mix A2 by pipetting up and down 3x and transfer 100 µl into A3
- [ ] Mix A3 by pipetting up and down 3x and transfer 100 µl into A4
- [ ] Mix A4 by pipetting up and down 3x and transfer 100 µl into A5
- [ ] Mix A5 by pipetting up and down 3x and transfer 100 µl into A6
- [ ] Mix A6 by pipetting up and down 3x and transfer 100 µl into A7
- [ ] Mix A7 by pipetting up and down 3x and transfer 100 µl into A8
- [ ] Mix A8 by pipetting up and down 3x and transfer 100 µl into A9
- [ ] Mix A9 by pipetting up and down 3x and transfer 100 µl into A10
- [ ] Mix A10 by pipetting up and down 3x and transfer 100 µl into A11
- [ ] Mix A11 by pipetting up and down 3x and transfer 100 µl into liquid waste
- [ ] TAKE CARE NOT TO CONTINUE SERIAL DILUTION INTO COLUMN 12.
- [ ] Repeat dilution series for rows B, C, D
- [ ] Measure fluorescence of all samples in all standard measurement modes in instrument
- [ ] Other: ____________________

27. **Part 2 (Cuvette Protocol): Prepare the serial dilutions of FITC**

If you are using cuvettes, please follow this protocol. You will need 11 (eleven) 2.0mL tubes (or larger) to make your serial dilution. Please check off each step that you followed. If you did anything differently or extra, please note that in the "Other" box.

Check all that apply.

- [ ] Add 1 mL of PBS into tubes 2-11
- [ ] Add 2.0 mL of FITC 1x solution tube 1
- [ ] Transfer 100 µl of FITC 1x solution from A1 into A2.
- [ ] Mix tube 2 by pipetting up and down 3x and transfer 1 mL into tube 3…
- [ ] Mix tube 3 by pipetting up and down 3x and transfer 1 mL into tube 4…
- [ ] Mix tube 4 by pipetting up and down 3x and transfer 1 mL into tube 5…
- [ ] Mix tube 5 by pipetting up and down 3x and transfer 1 mL into tube 6…
- [ ] Mix tube 6 by pipetting up and down 3x and transfer 1 mL into tube 7…
- [ ] Mix tube 7 by pipetting up and down 3x and transfer 1 mL into tube 8…
- [ ] Mix tube 8 by pipetting up and down 3x and transfer 1 mL into tube 9…
- [ ] Mix tube 9 by pipetting up and down 3x and transfer 1 mL into tube 10…
- [ ] Mix tube 10 by pipetting up and down 3x and transfer 1 mL into tube 11…
- [ ] Mix tube 11 by pipetting up and down 3x and and transfer 1 mL into liquid waste
- [ ] Measure fluorescence of all samples in all standard measurement modes in instrument
- [ ] Other: ....

28. **Step 3. Measurement** *

Please check off each step that you followed. If you did anything differently or extra, please note that in the "Other" box.

Check all that apply.

- [ ] Measure fluorescence of all of your samples.
- [ ] Import data into "Fluorescence" blue cells in provided Excel calibration sheet
- [ ] Other: ....

Skip to question 29.

Cell measurement protocol

Prior to performing the measurement on the cells you should perform the calibration measurements. This will ensure that you understand the measurement process and that you can take the cell measurements under the same conditions.

Workflow for cell measurement

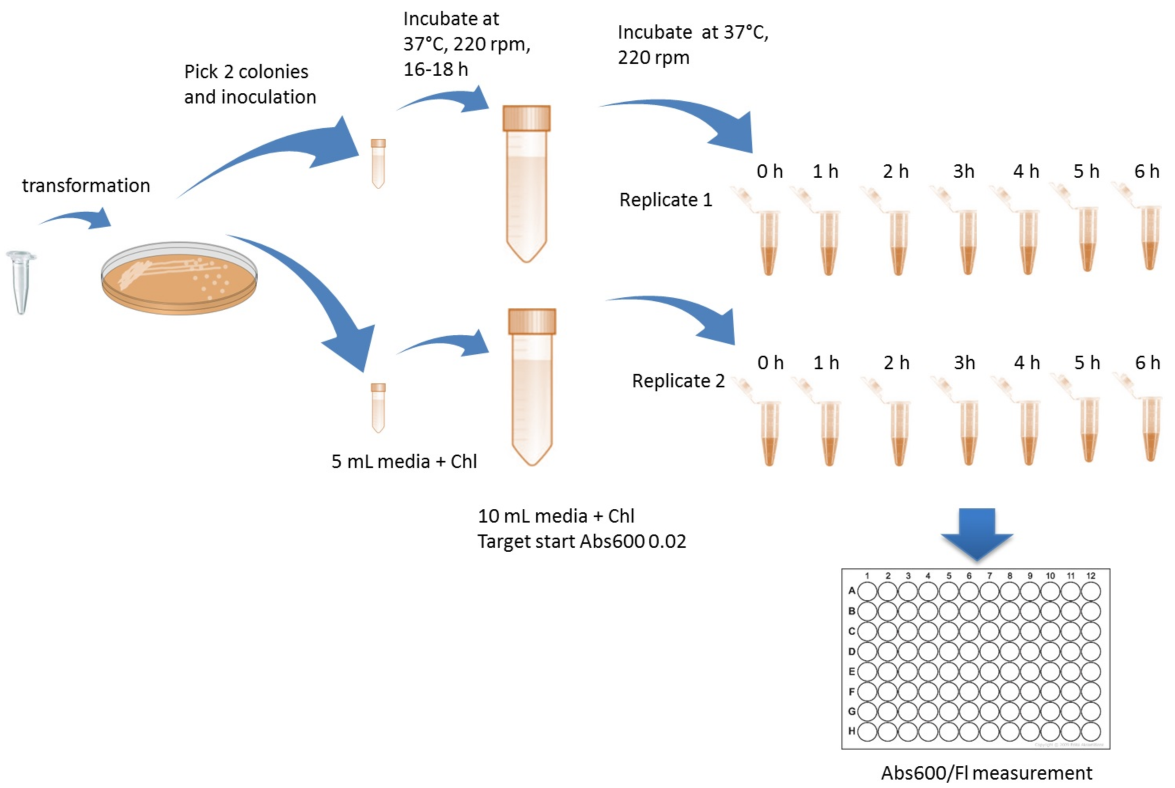

29. **Day 1 : Transformation** *

Transform Escherichia coli DH5α or TOP10 with these following plasmids (each plasmid in a different sample). Please check off each step that you followed. If you did anything differently or extra, please note that in the "Other" box.

Check all that apply.

- [ ] Positive control
- [ ] Negative control
- [ ] Device 1: J23101+I13504
- [ ] Device 2: J23106+I13504
- [ ] Device 3: J23117+I13504
- [ ] Other: ______________________

30. **Day 2 : Cell growth** *

Please check off each step that you followed. If you did anything differently or extra, please note that in the "Other" box.

Check all that apply.

- [ ] Pick 2 colonies from each of plate and inoculate it on 5-10 mL LB medium + Chloramphenicol (For antibiotic concentrations, please follow these guidelines: http://parts.igem.org/Help:Protocols/Antibiotic_Stocks).
- [ ] Grow the cells overnight (16-18 hours) at 37°C and 220 rpm.
- [ ] Other: ______________________

31. **Continue: Did you set up biological replicates in duplicate? ***

Biological replicates are where different samples that are expected to be identical are measured. This informs you about the variability across your organisms that contain the same device. For example, if you are using E. coli, this would be done by measuring the fluorescence from 2 different colonies containing the same device.

Mark only one oval.

- ( ) Yes
- ( ) No

32. **Day 3 : Cell growth, sampling, and assay ***

Please check off each step that you followed. If you did anything differently or extra, please note that in the "Other" box.

Check all that apply.

- [ ] Set your instrument to read OD600 (as OD calibration setting)
- [ ] Measure OD600 of the overnight cultures
- [ ] Import data into blue cells in Excel (normalisation) sheets provided
- [ ] Dilute the cultures to a target OD600 of 0.02 (see the volume of preloading culture and media in Excel (normalisation) sheets) in 10 ml 0.5x TB medium + Chloramphenicol in 50 mL falcon tube (if using cuvettes, you can use 100 ml in a 500 ml shake flask).
- [ ] Incubate the cultures at 37°C and 220 rpm.
- [ ] Take 100 µL (1% of total volume) cultures at 0, 1, 2, 3, 4, 5, and 6 hours of incubation (if using cuvettes, remove 1 ml from 100 ml culture).
- [ ] Place samples on ice.
- [ ] Other: ....

33. **What is the initial OD600 measurement of your overnight cultures? ***

Please list all devices and replicate number (if applicable) with their OD600 next to them.

....

....

....

....

....

34. **What type of media did you use for this step? ***

For E. coli, we recommend Terrific broth (at half strength: 0.5x TB) or can use LB (Luria Bertani) media as an alternative supplemented with the appropriate antibiotic. For antibiotic concentrations, please follow these guidelines: http://parts.igem.org/Help:Protocols/Antibiotic_Stocks

Mark only one oval.

- ( ) 0.5x Terrific Broth
- ( ) Luria Bertani
- ( ) Other: ....

35. **What type of vessel or container did you use to grow your cells?** *

Mark only one oval.

- 50 ml Falcon tube
- 500 ml shake flask
- Other: ....

Recommended 96-well plate layout for Abs600 and Fluorescence measurement

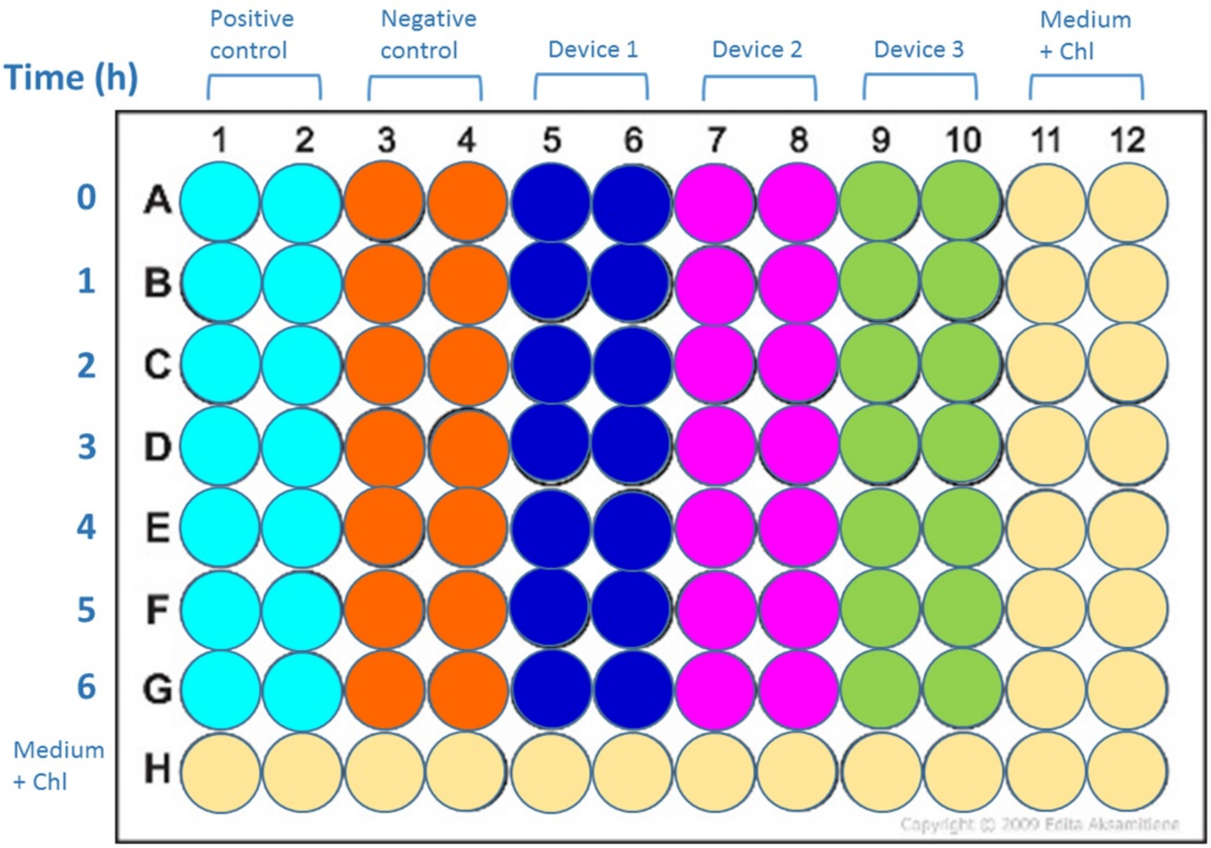

36. **What temperature setting did you use during the measurement?**

....

37. **Measurement** *

It is important that you use the same instrument settings that you used when measuring the FITC standard curve. This includes using the same sample volume (e.g., 100 µl) for measurement using the spectrophotometer. Samples should be laid out according to the figure layout for Abs600 and Fluorescence measurement. Pipette 100 µl of each sample into each well. Set the instrument settings as those that gave the best results in your calibration curves (no measurements off scale). If necessary you can test more than one of the previously calibrated settings to get the best data (no measurements off scale). Please check off each step that you followed. If you did anything differently or extra, please note that in the "Other" box.

Check all that apply.

- [ ] Measure OD and fluorescence of all samples
- [ ] Import data into blue cells in Excel (cell measurement) sheets provided
- [ ] Other: ....

Additional

38. **Additional information**

If you need more space to explain your growth conditions, please use the box below. We would expect this for mammalian work or non-traditional bacterial chassis - but all teams are welcome to add more details.

....
....
....
....
....

39. **Alternate Protocol or Additional Details**

Did you follow a different protocol? Please describe your protocol below. You may also add more details below if you followed our protocol.

....
....
....
....
....

Feedback

40. **Please rate your experience with filling in this InterLab Protocol form.** *

Mark only one oval.

- ( ) Very easy to fill in, no problems
- ( ) Took a long time to fill out, but was easy to understand
- ( ) Did not understand one or two questions
- ( ) Did not understand an entire section
- ( ) Very difficult to use; numerous problems
- ( ) Other: ....

41. **Please let us know any other thoughts or comments you have about the InterLab study experience.**

....

....

....

....

....

Powered by
Google Forms
