# Supplementary material for: Quantification of bacterial fluorescence using independent calibrants
Source: PLoS One. 2018 Jun 21;13(6):e0199432. doi: 10.1371/journal.pone.0199432 (PMC6013168; doi:10.1371/journal.pone.0199432)
Supplement: S3 File — Detailed protocol specification and reporting form provided for flow cytometers for the 2016 iGEM Interlab Study. (PDF) [file pone.0199432.s003.pdf]

Edit this form

2016 InterLab Worksheet for Flow Cytometry

Every team that participates in the 2016 iGEM InterLab study needs to fill in this form.

IMPORTANT: If you measured your devices using multiple pieces of equipment, please fill out this form for each different type of measurement you obtained.

If you have any questions or problems filling in this form, please email us at measurement at igem dot org.

* Required

Team name *

Please enter your team name below.

InterLab Study wiki page *

Please provide the direct link to your team's wiki page for the InterLab study. As explained in the InterLab requirements, all teams must have a specific page for the InterLab study on their wiki.

Individuals responsible for conducting InterLab study *

Please list everyone involved with creating the devices, measuring them, and processing the data. Please indicate which role each person filled. List anyone else who should be credited, e.g., in a publication based on this data.

Date of InterLab Study *

Please note the date(s) that the measurement was obtained.

Did your team participate in the Extra Credit? *

Details for the Extra Credit in 2015 can be found here:
http://2016.igem.org/Tracks/Measurement/Interlab_study

- ◯ Yes
- ◯ No

If you participated in Extra Credit, please enter a link to a page on your team wiki that describes your extra credit work *

Equipment Information

What type of incubator did you use to grow your cells? *

Please provide as much information as possible in terms of the equipment type, name, and model.

If known, what was your incubator's throw (shaking diameter)?

This information is often found with the manufacturer's specifications for the incubator.

What model of flow cytometer did you use to measure the devices? *

Please provide as much information as possible in terms of the equipment type, name, model, and any custom modifications that have been made to the instrument.

When was this equipment last calibrated? *

Who calibrated the equipment? *

What was the wavelength of light you used to excite the cells? *

The recommended wavelength for GFP is a 488nm laser

What was the filter on the channel you used to capture the light emission from the cells? *

The recommended filter for GFP is 530/30 (meaning a bandpass filter with 530 nm center, 30 nm width)

What quantity was recorded for each event? *

Flow cytometers may report "area", "height" and/or "width" of each event. If possible, you should report area, which will often be indicated by a "-A" in the channel name, e.g., "FITC-A." The reason to favor area is that it gives the total fluorescence observed for each particle, whereas height gives maximum fluorescent intensity and width gives the length the particle was observed.

- Area
- Height
- Width
- Other:

If you have other information or details you wish to provide about your equipment, please use the box below.

Prepare Cells for Flow Cytometry

Day 1 : Transformation (or streak plate) *

Transform Escherichia coli DH5α or TOP10 with these following plasmids (each plasmid in a different sample). Please check off each step that you followed. If you did anything differently or extra, please note that in the "Other" box. Likewise, if you have already transformed and are streaking a fresh plate for colonies, note that in the "Other" box.

- Positive control
- Negative control
- Device 1: J23101+I13504
- Device 2: J23106+I13504
- Device 3: J23117+I13504
- Other:

Day 2 : Cell growth *

Please check off each step that you followed. If you did anything differently or extra, please note that in the "Other" box.

- Pick 3 colonies from each of plate and inoculate it on 5-10 mL LB medium + Chloramphenicol (For antibiotic concentrations, please follow these guidelines: http://parts.igem.org/Help:Protocols/Antibiotic_Stocks).
- Grow the cells overnight (16-18 hours) at 37°C and 220 rpm.

- [ ] Other: ______

Continue: Did you set up biological replicates in triplicate? *

Biological replicates are where different samples that are expected to be identical are measured. This informs you about the variability across your organisms that contain the same device. For example, if you are using E. coli, this would be done by measuring the fluorescence from 3 different colonies containing the same device.

- [ ] Yes
- [ ] No

Day 3 : Cell growth, sampling, and assay *

Please check off each step that you followed. If you did anything differently or extra, please note that in the "Other" box. We recommend you read the OD600 of your culture, if possible. Please note the instrument you used to measure OD600 in the "Other" box if you were able to do so.

- [ ] Set your instrument to read OD600 (as OD calibration setting)
- [ ] Measure OD600 of the overnight cultures
- [ ] Dilute the cultures to a target OD600 of 0.02 (see the volume of preloading culture and media in Excel (normalisation) sheets) in 10 ml 0.5x TB medium + Chloramphenicol in 50 mL falcon tube (if using cuvettes, you can use 100 ml in a 500 ml shake flask).
- [ ] Incubate the cultures at 37°C and 220 rpm.
- [ ] Take 100 µL (1% of total volume) cultures at 6 hours of incubation (if using cuvettes, remove 1 ml from 100 ml culture).
- [ ] Dilute cells to the appropriate density for your flow cytometer. If unknown, check with the person who maintains the instrument.
- [ ] Measure cells using flow cytometer.
- [ ] Other: ______

What is the initial OD600 measurement of your overnight cultures? *

Please list all devices and replicate number (if applicable) with their OD600 next to them.

What type of media did you use for this step? *

For E. coli, we recommend Terrific broth (at half strength: 0.5x TB) or can use LB (Luria Bertani) media as an alternative supplemented with the appropriate antibiotic. For antibiotic concentrations, please follow these guidelines: http://parts.igem.org/Help:Protocols/Antibiotic_Stocks

- ○ 0.5x Terrific Broth
- ○ Luria Bertani
- ○ Other: ______

What type of vessel or container did you use to grow your cells? *

- ○ 10 ml culture tube
- ○ 50 ml Falcon tube

- ◯ 500 ml shake flask
- ◯ Other: ________

Acquire Data with Flow Cytometer

Please check off each step that you followed. If you did anything differently or extra, please note that in the "Other" box. *

- ☐ Adjust side-scatter (SSC) and forward scatter (FSC) PMT voltages using bacteria from your negative control, until the distribution of each is centered on the scale.
- ☐ Adjust FITC/GFP PMT voltage using bacteria from your positive control, until the upper edge of the “bell curve” from the fluorescent population is one order of magnitude below the upper end of the scale.
- ☐ Acquire at least 10,000 events from a sample of calibration beads.
- ☐ Acquire at least 10,000 events for each biological sample.
- ☐ Other: ________

Calibration to Standard Units

Please check off each step that you followed. If you did anything differently or extra, please note that in the "Other" box. If you have followed the rest of this protocol, you should be able to use the “flow_cyometry_workbook” Excel file to make the unit conversion calculations. *

- ☐ Examine a histogram of the RCP-30-5A beads and find the observed centers of at least the three most strongly fluorescent large peaks. See the provided example file.
- ☐ Divide the MEFL value for each RCP-30-5A peak by the observed peak centers, to produce a conversion ratio.
- ☐ Take the average of the conversion ratios: multiplying arbitrary units by this mean conversion ratio will change them into MEFL.
- ☐ Compute the geometric mean of fluorescence for each biological sample, excluding all events with values below 10.
- ☐ Multiply the geometric mean fluorescence for each sample by the mean conversion ratio, to produce a value in MEFL.
- ☐ Other: ________

Report Results

Please fill in the provided Excel file and email it to measurement@igem.org. If you cannot use the Excel file, please use another spreadsheet program or email the measurement@igem.org to arrange how best to contribute your data.

Feedback

Please rate your experience with conducting the InterLab Study *

- ◯ Very easy to participate; little to no problems
- ◯ Cloning problems made it more difficult than expected
- ◯ Equipment problems made it more difficult than expected
- ◯ Instructions were unclear and made it difficult to participate

- ◯ Very difficult to participate; numerous problems
- ◯ Other: ______

Please rate your experience with filling in this InterLab Worksheet *

- ◯ Very easy to fill in, no problems
- ◯ Took a long time to fill out, but was easy to understand
- ◯ Did not understand one or two questions
- ◯ Did not understand an entire section
- ◯ Very difficult to use; numerous problems
- ◯ Other: ______

Please let us know any other thoughts or comments you have about the InterLab study experience.

Submit

Never submit passwords through Google Forms.

100%: You made it.

Powered by
Google Forms

This content is neither created nor endorsed by Google.
Report Abuse - Terms of Service - Additional Terms
